# Primary Breast Leiomyosarcoma

**DOI:** 10.1155/2013/732730

**Published:** 2013-12-22

**Authors:** L. Amaadour, Z. Benbrahim, K. Moumna, L. Boudahna, A. Amarti, S. Arifi, N. Mellas, O. El Mesbahi

**Affiliations:** ^1^Department of Medical Oncology, HASSAN II University Hospital, Fez, Morocco; ^2^Department of Pathology, HASSAN II University Hospital, Fez, Morocco

## Abstract

Primary leiomyosarcoma of the breast is an extremely rare neoplasm. Only few cases have been reported in the literature. We report here a case of breast leiomyosarcoma in a 44-years-old female and we discuss the data of the existing literature.

## 1. Introduction

Breast sarcomas are rare nonepithelial malignancies that arise from the connective tissue within the breast [1]. They constitute about 1% of all breast malignancies [2]. Leiomyosarcoma subtype remains the least frequent. The average age of occurrence is ranging between 45 and 50 years [2–4]. In this case report, we present the clinical features of a 44-year-old woman with primary leiomyosarcoma of the breast, its pathological and therapeutic outcomes, and an up-to-date review of the literature on the topic.

## 2. Case Presentation

**Figure 1:**
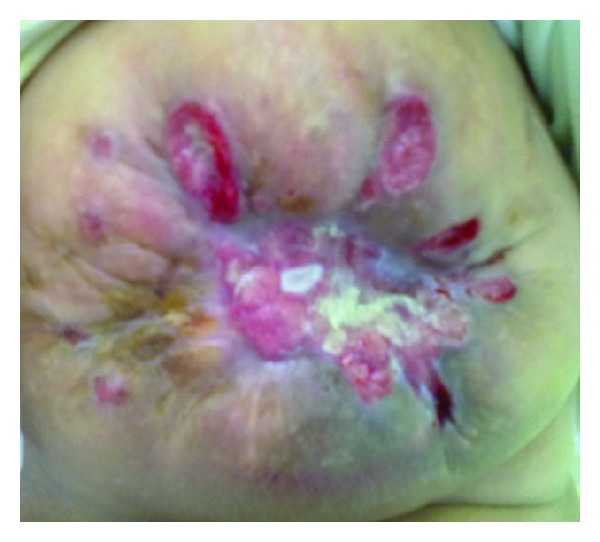
Photograph showing lobulated and ulcerative breast tumor.

**Figure 2:**
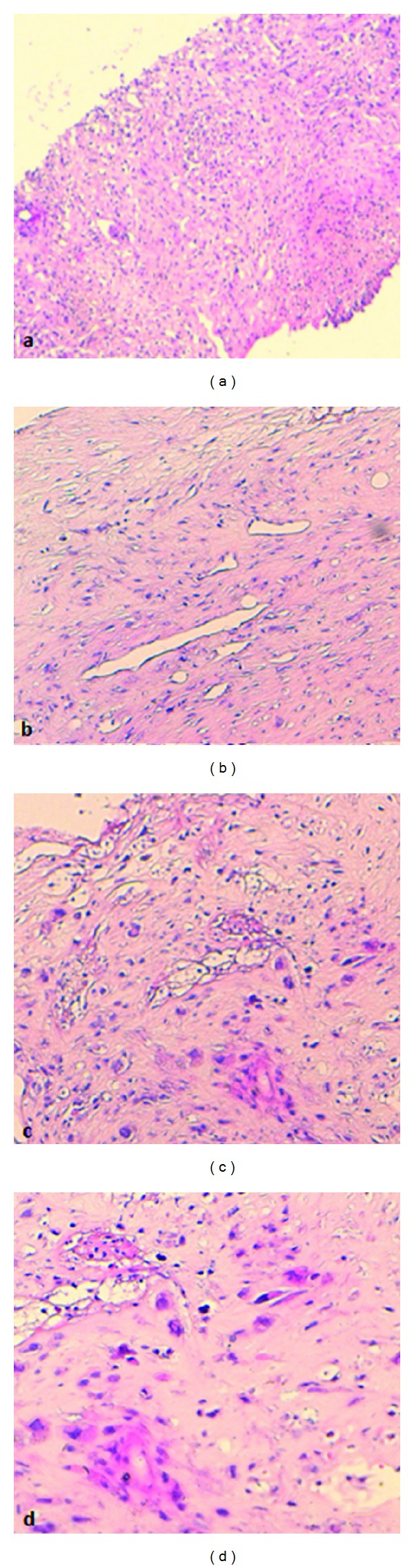
(a), (b), (c), and (d) Progressive increasing magnification of histology (HES 5, 10, 10, and 20, resp.) showing a tumor composed of hyperchromatic spindle cells with frequent atypical nuclei.

**Figure 3:**
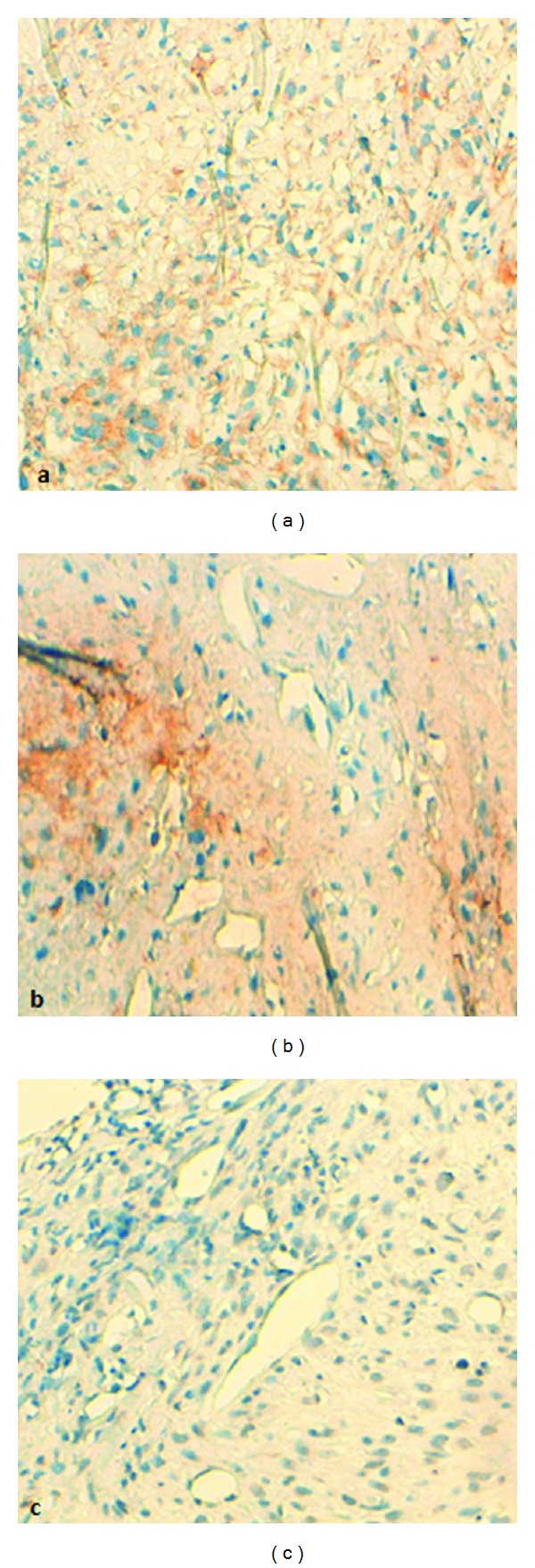
(a), (b) Immunochemistry positivity for desmin and H-caldesmon, respectively. (c) Negative immunohistochemical reactivity for cytokeratin.

A 44-year-old Arab woman presented with a four-month history of rapidly increasing painless mass in her right breast. On clinical examination, the mass was lobulated and ulcerative taking almost all of the mammary gland and involving breast skin and the nipple areola (Figure 1). No axillary lymph nodes were palpable. An ultrasonography of the breast identified a hypoechoic and heterogeneous mass with an axillary lymphadenopathy measuring 9.2 cm × 7.6 cm × 6 cm and 1.3 cm, respectively. Core needle biopsy of the mass was performed. Pathology revealed tumor comprised of hyperchromatic spindle cells arranged in fascicles, with marked pleomorphism, atypical nuclei, and a moderate mitotic activity (6/10 high power field) (Figure 2). Immunohistochemistry showed that the tumor cells were positive for desmin and H-caldesmon while they were negative for cytokeratin (Figure 3).

The extent of disease was evaluated by systemic physical examination and chest and abdominopelvic computed tomography which revealed diffuse pulmonary metastasis.

**Figure 4:**
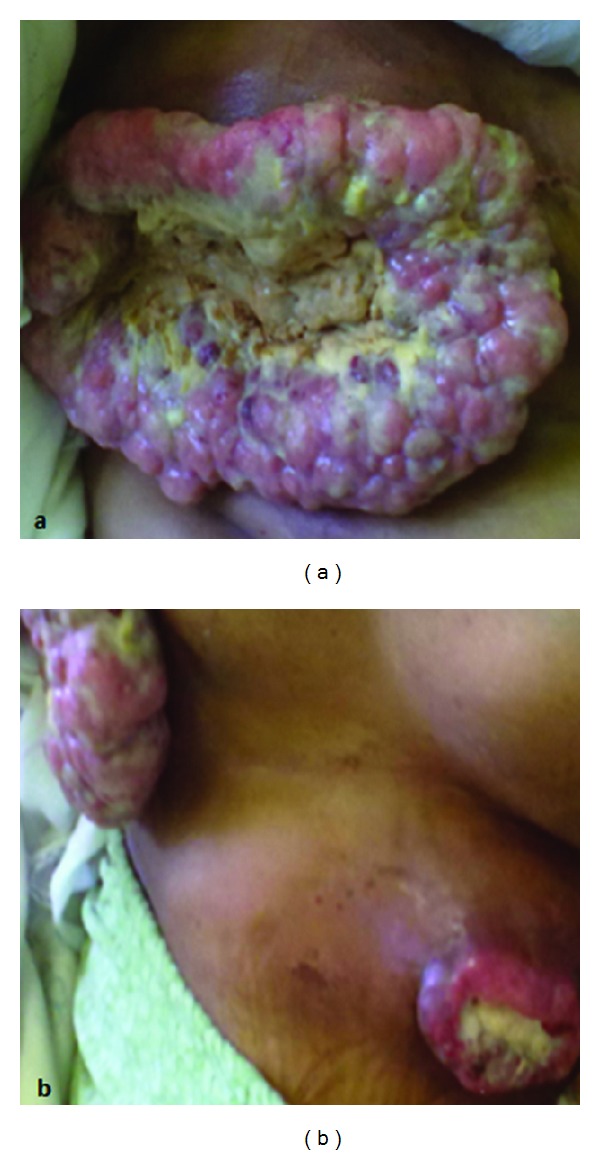
(a) Photograph of local progression of the breast tumor after 3 cycles of the first line chemotherapy and distant apparition of a cutaneous abdominal nodule.

Palliative chemotherapy based on doxorubicin (60 mg/m²) and ifosfamide (9 g/m²) every 3 weeks was indicated. After three cycles of this regimen, we noted a clear local and distant progression including the increase in the size of the mammary tumor and the occurrence of a well-circumscribed and ulcerative cutaneous nodule in the abdominal wall (Figure 4). Second line docetaxel (75 mg/m² day 8)—gemcitabine (900 mg/m² days 1 and 8) regimen was proposed, but the patient died of disease one month later.

## 3. Discussion

Leiomyosarcomas of the breast are uncommon neoplasms. The exact origin of these tumors is debated. They can arise either from the smooth muscle cells lining blood vessels or from stromal mesenchymal cells [5, 6]. Breast sarcoma often presents as palpable and well-circumscribed large tumors [3, 7]. The presence of clinical lymphadenopathy is exceptional, occurring in less than 10% [8, 9]. Breast skin and the nipple areola are rarely involved [10, 11], unlike our case where the tumor was very locally advanced involving the entire gland. Findings on mammography and ultrasound are nonspecific [12]. Core biopsy is the diagnostic procedure of choice if sarcomas are suspected [13]. Immunohistochemical staining is essential as adjuncts to differentiate leiomyosarcomas from other tumors and soft tissue sarcomas. These tumors are usually positive for desmin, smooth muscle actin, and muscle specific actin and negative for S100, cytokeratins, and epithelial markers [14, 15].

The limited number of cases treated at any one institution has hampered making specific recommendations regarding surgery and medical treatments for this pathological entity. Otherwise, treatment principles have been extrapolated from studies of nonbreast soft tissue sarcomas. Certainly, the cornerstone of treatment is complete excision with negative margins [11], although there is no consensus on the use of adjuvant chemotherapy or radiotherapy [16]. Patients with metastatic breast sarcoma are offered palliative chemotherapy; palliative surgery is proposed when local complications from primary breast sarcomas cause significant morbidity [17, 18]. However isolated and controlled metastatic disease may also be amenable to potentially curative resection [18, 19].

Most of the cases reported till date of primary breast leiomyosarcoma have undergone mastectomy without adjuvant treatment [20]. To the best of our knowledge, our patient is one of the rare cases of primary breast leiomyosarcoma to be diagnosed in an advanced stage of disease [21].

The anthracyclines based chemotherapy is the standard first line treatment [22]. Therefore, multiagent chemotherapy with adequate-dose anthracyclines plus ifosfamide may be indicated to offer advantage of potentially curative metastasectomy in patients with isolated pulmonary metastasis [19]. After failure of the first line chemotherapy, therapeutic options include gemcitabine alone or in association with dacarbazine or docetaxel [23, 24].

## 4. Conclusion

Primary leiomyosarcoma of the breast is a diagnostic challenge to clinicians as it is a rare entity which has no specific clinical or radiological features. A general agreement on treatment is still lacking, since there are no prospective trials to guide therapy.
